# Primary renal Ewing sarcoma in a postpartum female: a case report

**DOI:** 10.3389/fonc.2025.1600958

**Published:** 2025-09-04

**Authors:** Xiaohui Feng, Xiaomei Wang, Feng Gao

**Affiliations:** Department of Pathology, Hebei Medical University Third Hospital, Shijiazhuang, China

**Keywords:** Ewing sarcoma, pregnancy, kidney, histopathology, immunohistochemistry

## Abstract

Primary renal Ewing sarcoma (rES) is an extremely rare and aggressive malignancy in adults with nonspecific clinical and imaging features. The diagnosis of rES during pregnancy is particularly uncommon. Here, we report a unique case of rES in a 35-year-old postpartum female who presented with lumbar pain and hematuria. Histopathological and immunohistochemical analyses confirmed the diagnosis of rES. This case highlights the importance of considering rES in young females with renal masses, particularly during the postpartum period.

## 1 Introduction

Ewing sarcoma family tumors (ESFTs) are a group of tumors originating from the neuroectoderm with similar morphology and expression of the ectopic fusion gene EWS/FLI-1. This group includes Ewing sarcoma (ES), peripheral primitive neuroectodermal tumors (PNET), extraosseous ES, and skin tumors (chest lung PNET) (1). These tumors are highly malignant, composed of small round cells, commonly seen in children and adolescents, and rare in adults. The limbs, trunk, soft tissues of the head and neck, and retroperitoneum are common sites of extraosseous ES, accounting for only 6% of ESFT (2). ES, which occurs in the kidneys, is rare. Primary renal ES (rES) clinically presents with lower back pain, hematuria, and rapidly growing masses. The tumor exhibits highly aggressive biological behavior and is prone to early metastasis to the lungs, bones, and lymph nodes, resulting in a poor prognosis (3, 4). This study aimed to report on a postpartum patient with rES by analyzing the imaging and histomorphological characteristics of the rES and reviewing the published literature.

## 2 Case description

**Figure 1:**
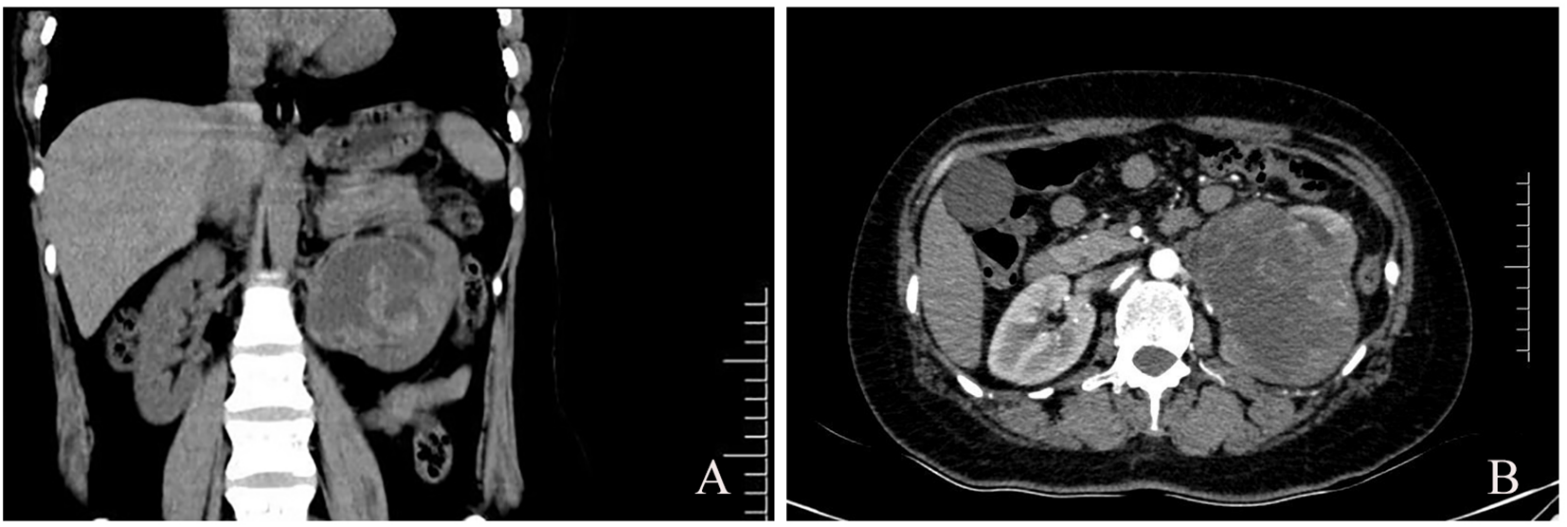
Abdominal CT. **(A)** Coronal view showing the tumor located in the middle of the kidney, occupying the position of the renal pelvis. **(B)** Axial view of contrast-enhanced CT demonstrating a large heterogeneously enhancing solid-cystic mass, measuring 9.32×7.46×6.24cm. No renal vein tumor thrombosis was observed.

A 35-year-old woman complained of bilateral lower back discomfort for 2 months, accompanied by a feeling of heaviness and bloating in the lower abdomen. She had no fever, dysuria, or weight loss. One month earlier, a urine test showed occult blood, with subsequent transient gross hematuria. She was admitted to our hospital for urothelial carcinoma. Initial computed tomography (CT) scan revealed a large mass in the left kidney. Enhanced CT scan demonstrated a large cystic solid mass, with inhomogeneous density and size of about 9.32×7.46×6.24cm. The mass was located in the renal parenchyma and protruded into the renal pelvis. The renal pelvis was locally dilated, and the renal parenchyma appeared thinner and less dense. The left renal vein was compressed and displaced without filling defects. No invasion of the renal veins or inferior vena cava was observed (**Figure 1**). Further CT imaging of the chest and abdomen showed no metastasis to the lung or liver. Laboratory tests showed anemia (hemoglobin111.10g/L, normal range:115–150 g/L) and abnormalities in blood coagulation: prolonged prothrombin time (13.6 s, normal range), reduced prothrombin activity (73%, normal range:80%-160%), increased fibrinogen degradation products (10.77μg/mL, normal range:0-5μg/mL), and decreased D-dimer (4.72μg/mL, normal range:0-0.5μg/mL). Other blood tests, including serum creatinine levels, showed no significant abnormalities.

**Figure 2:**
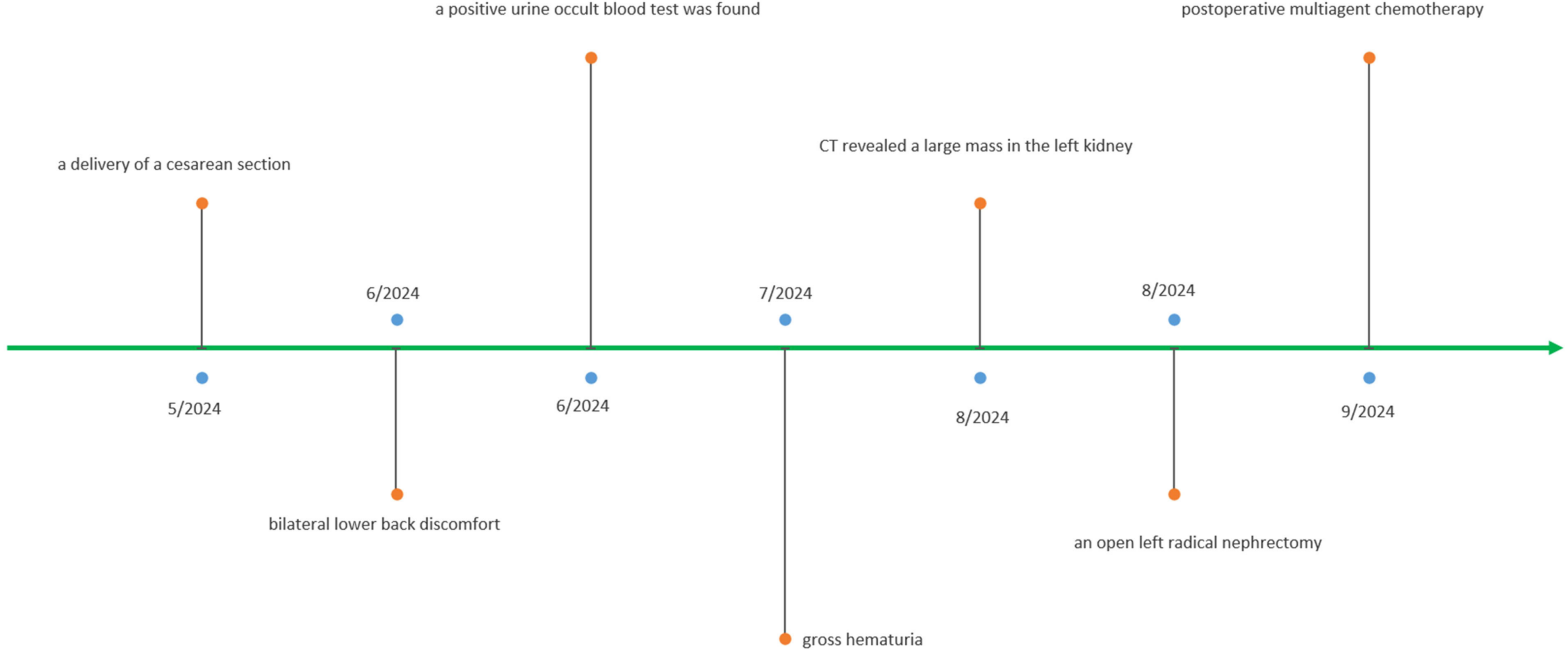
Timeline of the patient’s disease course.

The patient underwent a cesarean section just one month before the onset of clinical symptoms, with no abnormalities throughout pregnancy. She had no significant medical history related to the tumor. Open left radical nephrectomy was performed (**Figure 2**). Grossly, the kidney measured 11.5×7.5×5cm. Within the mid-portion of the kidney, a tumor measuring 7×5×4 cm showed a solid, fish-flesh appearance on sectioning, accompanied by focal hemorrhagic and necrotic areas. Histopathological examination revealed:(a) At scanning magnification, a diffusely infiltrating tumor composed of solid sheets of cells interspersed with hemorrhagic and necrotic foci, resulting in compressive atrophy of adjacent renal tubules. (b) Higher-power views demonstrated monotonous small round cells with round to oval nuclei, finely dispersed chromatin, inconspicuous nucleoli, and scant cytoplasm.

**Figure 3:**
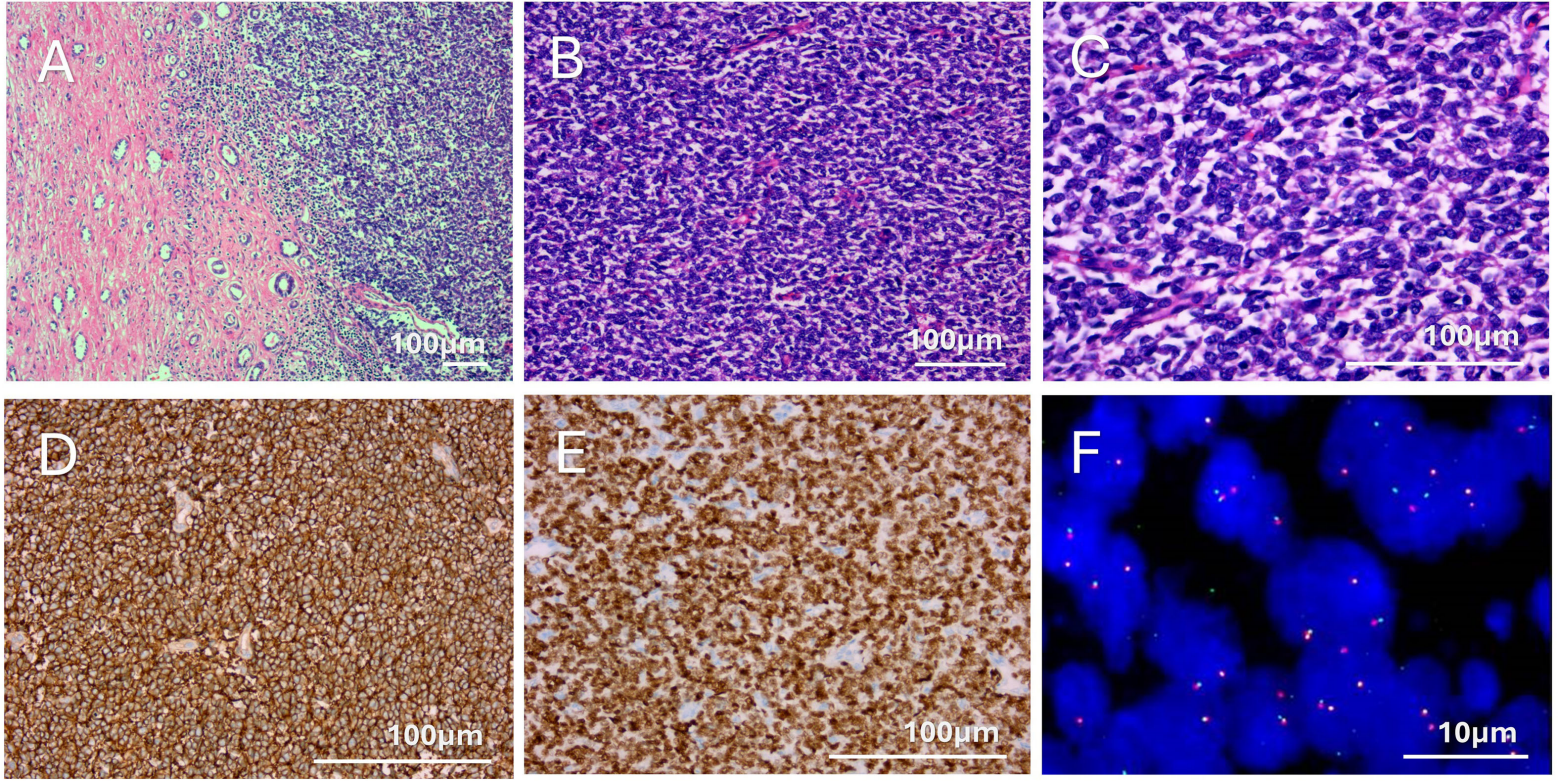
Histopathology and immunohistochemistry. **(A)** Malignant tumor component (right half) with areas of renal tubules (H&E, 10x). **(B)** Sheets of uniform round to oval tumor cells (H&E, 20x). **(C) **Tumor cells had uniform round to oval hyperchromatic nuclei, small nucleoli, and inconspicuous to scant cytoplasm (H&E, 40x). **(D)** Diffuse membranous CD99 staining (40x). **(E)** Malignant tumor cells showing diffuse nuclear NKX2.2 expression (40x). **(F)** FISH performed with break-apart EWSR1 probes shows a split signal pattern due to a rearrangement in the EWSR1 gene (100x).

Immunohistochemistry revealed that the tumor cells were diffusely positive for NKX2.2, vimentin, and CD99, with positive staining for CD56 and Ki-67 (50% positive). Other markers, including CA9, CD20, CD3, Pax-8, GATA3, p63, p40, SS18-SSX, S100, WT1, SOX10, Desmin, CK, CgA, INSM1, and FLI-1, were negative. These immunohistochemical findings led to the pathological diagnosis of primary rES. The diagnosis was confirmed by fluorescence *in situ* hybridization (FISH) analysis using an EWSR1 break-apart probe, which was positive (**Figure 3**).

The patient received postoperative multiagent chemotherapy and remained disease-free at the most recent follow-up 5 months after surgery.

## 3 Discussion

Renal cell carcinoma is the most common malignant neoplasm of the kidney, accounting for more than 90% of all kidney tumors. Renal sarcomas constitute less than 1% of all malignant renal tumors (5). rES is extremely rare and occurs in a younger age group (mean age, 30.4 years), with a slight male predominance (6). The overall 5-year disease-free survival rate ranges from 45% to 55% (7). The mean survival is 26.14 months, with lower median survival in patients with advanced metastatic disease (6). The most common sites of metastasis are the lungs and liver, followed by the abdominal lymph nodes and bones (8). Clinical symptoms of rES are nonspecific and include abdominal pain or lumbago, hematuria, an abdominal mass, fever, and weight loss. Imaging findings are also nonspecific. CT findings of rES show an inhomogeneous density of the tumor because of cystic necrosis and hemorrhage. During a dynamic enhanced scan, the tumor presents progressive and “septum-like” enhancement (9). Literature reports indicate that in one-third of the cases, rES invades the renal vein or inferior vena cava, forming a thrombus (10). However, this did not occur in our case.

Histopathological and immunohistochemical staining are important for the diagnosis of rES. Morphologically, it appears as sheets of a single small blue round cell, mimicking other tumors, such as Wilms tumor, lymphoma, rhabdomyosarcoma, small cell carcinoma, and monomorphic synovial sarcoma. Immunohistochemical staining can help differentiate rES from those tumors. NKX2.2, CD99, and vimentin are positive in rES, whereas LCA, Syn, CK, and MyoD1 are negative. Wilms tumors usually strongly express WT1 and fail to express membranous CD99, FLI-1, or nuclear NKX2.2. Lymphoma is positive for LCA, CD20, and CD3 but negative for NKX2.2. Rhabdomyosarcoma expresses desmin, MyoD1, or myogenin due to skeletal muscle differentiation, whereas small cell carcinoma expresses CK and CgA with or without Syn. Both Rhabdomyosarcoma and small cell carcinoma lack CD99 positivity. Synovial sarcomas show immunoreactivity for EMA, TLE1, and Bcl2 and lack expression of FLI-1 and NKX2.2 (10). Our case expressed NKX2.2, CD99, and vimentin, consistent with the reported findings. Our diagnosis also excluded sarcomatoid transformation of renal cell carcinoma and urothelial carcinoma through CA9, Pax-8, GATA3, P63, and P40. FISH and reverse transcription polymerase chain reaction can also be used for diagnosing rES because of the characteristic gene transformation t(11,22) (q24;q12) and fusion gene EWS-FLI (11).

Our patient exhibited no abnormal symptoms during gestation. She noticed slight back pain 1 month after giving birth and microscopic hematuria when she underwent a routine 42-day postpartum examination. Gross hematuria soon developed, and CT revealed an abdominal mass. She was initially admitted to our hospital for urothelial carcinoma; however, she was later diagnosed with rES, a rare entity. Pregnancy-associated primary rES is uncommon. To date, only five patients have been diagnosed with rES during pregnancy or the postpartum period (12–16). The clinical features of these patients are presented in **Table 1**. All were younger females. The tumor remains asymptomatic until it becomes large enough to produce symptoms, and the average tumor size at the time of diagnosis varies from 5 to 16 cm. Despite active treatment, two patients with distant metastasis died during follow-up (less than 2 years). The presence of metastasis correlated with adverse prognosis in both cases.

**Table 1:** Clinical features of patients with rES.

Case	Author and Year	Age (years)	History	Diagnosis in pregnancy/postpartum	Tumor size (cm)	Metastasis	Treatment	Follow-up	Status
1	Ding Y et al., 2016 (15)	21	N	5mo	15	Spleen, diaphragm, and lung	RN and chemo	15 mo	Dead
2	Miao C et al., 2018 (12)	40	N	6–7mo	5	No	RN	N	Alive
3	Babapour S et al., 2020 (13)	35	Megaloblastic anemia and pulmonary hypertension	Postpartum1mo	16	Lung and bone	RN and chemo	14 mo	Dead
4	Bray G et al., 2022 (14)	31	N	1mo	12	No	RN and chemo	12 mo	Alive
5	Curtin P et al.,2023 (16)	25	Addison’s disease, bilateral nephrolithiasis, and gestational diabetes	Postpartum 1mo	7	No	RN and chemo	24 mo	Alive

N, not mentioned; RN, radical nephrectomy; chemo, chemotherapy; mo, months.

Hormonal changes during pregnancy and the postpartum period may contribute to tumor development and progression. DuBois et al. (17) proposed that estrogen and/or progesterone do not exert a direct effect on tumor growth based on the limited expression of their receptors in ES cells. Pregnancy-associated plasma protein A (PAPP-A) is a protease primarily produced by the placenta during pregnancy. It mediates the cleavage of insulin-like growth factor-1(IGF-1) from its binding proteins. IGF-1 has been recognized as a critical growth factor in ES. Furthermore, a recent study identified overexpression of PAPP-A on the surface of tumor cells. PAPP-A promotes ES cell proliferation and survival by modulating IGF-1 bioavailability. In addition, PAPP-A enhances IGF-1 signaling, which indirectly suppresses tumor antigen presentation and facilitates immune evasion in ES (18). Nevertheless, additional studies are needed to determine the relationship between pregnancy and rES.

Diagnostic challenges in renal Ewing sarcoma (rES) during pregnancy or the postpartum period arise from several factors. The presenting symptoms—including low back pain, abdominal discomfort, and fatigue—are non-specific and often mimic common complaints during late pregnancy and the early postpartum period, potentially leading to delayed diagnosis. Radiological evaluation is further complicated by the strict limitation of abdominal CT imaging during pregnancy due to radiation risks. While ultrasound is a safer alternative, its sensitivity may be reduced for smaller renal masses owing to fetal obscuration or bowel gas interference. The rapid growth characteristic of rES enables lesions undetectable in mid-late pregnancy to manifest clinically within weeks postpartum. Consequently, the postpartum period represents a critical window for detection, during which routine postnatal examinations (e.g., the standard 6-week postpartum visit) provide opportunities for the incidental identification of renal tumors.

Owing to its rarity, a standard treatment for rES remains absent. A combination of radical nephrectomy, radiation therapy, and chemotherapy is the treatment modality for patients with early-stage disease. Radical nephrectomy, followed by chemotherapy is recommended (19). The literature reports poor prognostic factors, including the maximum tumor diameter (>10 cm), clinical symptoms of weight loss, metastasis at diagnosis, tumor thrombogenesis in the renal veins and/or inferior vena cava, and failure to undergo radical nephrectomy (9). As the tumor is highly aggressive and has a high mortality rate, it should not be ignored in young patients with renal masses, especially during pregnancy.

## Data Availability

The original contributions presented in the study are included in the article/supplementary material. Further inquiries can be directed to the corresponding author.
